# Factors Influencing Rate of Testicular Salvage in Acute Testicular Torsion at a Tertiary Pediatric Center

**DOI:** 10.5811/westjem.2014.11.22495

**Published:** 2015-01-07

**Authors:** Puneeta Ramachandra, Kerrin L. Palazzi, Nicholas M. Holmes, Sarah Marietti

**Affiliations:** * Valley Children’s Hospital, Madera, California; † University of California, San Diego, Moores Cancer Center, Division of Urology, La Jolla, California; ‡ Rady Children’s Hospital San Diego, Division of Pediatric Urology, San Diego, California

## Abstract

**Introduction:**

Studies have demonstrated that variables other than duration of symptoms can affect outcomes in children with acute testicular torsion. We examined demographic and logistical factors, including inter-hospital transfer, which may affect outcomes at a tertiary pediatric referral center.

**Methods:**

We reviewed charts of all pediatric patients with acute testicular torsion during a five-year period. Data were collected regarding age, insurance type, socioeconomic status, duration of symptoms prior to presentation, transfer status, time of day, time to surgical exploration, and testicular salvage.

**Results:**

Our study included 114 patients. Testicular salvage was possible in 55.3% of patients. Thirty-one percent of patients included in the study were transferred from another facility. Inter-hospital transfer did not affect testicular salvage rate. Time to surgery and duration of pain were higher among patients who underwent orchiectomy versus orchidopexy. Patients older than eight years of age were more likely to undergo orchidopexy than those younger than eight (61.5% vs. 30.4%, p=0.01). Ethnicity, insurance type, or time of day did not affect the testicular salvage rates. On multivariate analysis, only duration of symptoms less than six hours predicted testicular salvage (OR 22.5, p<0.001).

**Conclusion:**

Even though inter-hospital transfer delays definitive surgical management, it may not affect testicular salvage rates. Time to presentation is the most important factor in predicting outcomes in children with acute testicular torsion.

## INTRODUCTION

The management of the acute scrotum in pediatric patients can be challenging. In a patient with acute testicular torsion, a delay in presentation, diagnosis, or definitive management may result in poor outcomes such as loss of the affected testis. Previous studies have demonstrated that a number of variables can affect testicular salvage, including symptom duration, insurance type, and race.^1^–^3^ Age has been shown to negatively impact testicular salvage in some studies,^4^ while other series show a positive correlation with age and testicular salvage.^1^–^3^

In geographic areas where pediatric specialty care is unavailable, children may be transferred to tertiary care centers for emergent conditions. Reasons for transferring a pediatric patient for acute scrotum may include lack of availability of pediatric urologists or anesthesiologists, lack of appropriate diagnostic modalities, patient preference, insurance status, or other explanations. Transfer from one hospital to another inherently delays definitive management and may ultimately affect outcome.

The aim of this study was to examine the cohort of patients seen at a tertiary pediatric referral center, comparing patients who presented primarily to our facility to those who were transferred from another facility. We hypothesized that factors other than symptom duration, such as transfer from an outside facility, age, time of day of presentation, insurance type or ethnicity, may affect testicular salvage in patients with acute scrotum.

## METHODS

Following approval from the local institutional review board, we retrospectively reviewed charts of all patients with a diagnosis of acute testicular torsion presenting between 2005 and 2011. Charts to be abstracted were identified based on having an ICD-9 code for testicular torsion (608.2, 608.20, 608.21, 608.22). All charts were electronic. We included all patients seen in the emergency department (ED) who underwent surgical exploration at our institution. Our referral center is the main pediatric treatment facility for a large geographic area, routinely caring for patients who live more than two hours away. Patients who were transferred were sent from the outside facilities’ EDs to the ED at our facility, where they were reevaluated prior to surgery. A diagnosis of testicular torsion was made prior to surgical exploration and confirmed at the time of surgery. All patients underwent Doppler ultrasonography either at their presenting hospitals or at our institution prior to surgical exploration. We excluded from the analysis patients with neonatal torsion and suspected intermittent torsion who were treated in a non-emergent fashion.

All charts were reviewed and data extracted by a single reviewer (the lead author). Data were extracted from hospital charts regarding age, insurance type, ethnicity, whether or not the patient was transferred, presentation before 5pm vs. after 5pm, duration of symptoms prior to presentation in any ED, time from first presentation in any ED to surgical exploration, and results of scrotal exploration (orchiectomy vs. orchidopexy). We decided to use a cutoff time of 5pm to analyze patients, hypothesizing that the time the patient presented could influence the decision to transfer a patient to our facility. The decision to proceed with orchiectomy or orchidopexy was made by each individual surgeon based on the appearance of the testicle during exploration. Insurance type was categorized as Medicaid, private (PPO/HMO), or managed Medicaid (Medi-cal HMO). We excluded from the analysis seven patients with a particular type of insurance (CPCMG) as these patients are contractually obligated to be treated at our facility. Symptom duration was recorded as the longest duration of symptoms prior to initial presentation reported by the patient or caregiver to any provider during the evaluation. We characterized ethnicity as Hispanic or non-Hispanic. Records from transferring facilities were not available for review in all cases; therefore, we could not record the reason for transfer or whether or not a local urologist was contacted before initiating transfer. We calculated the time to surgical exploration as time from first presentation in any ED to the operating room start time recorded on the anesthesia record at our facility.

Univariate comparative statistics including Mann-Whitney u-test and Spearman Rank correlation for continuous, non-normally distributed variables, and Chi^2^ test and Fisher’s exact test for categorical variables, were used to examine associations between our variables of interest and testicular salvage. Age was found to have a bimodal distribution and was examined as both a continuous measure and grouped into younger than eight years vs. ≥ eight years-old. We used binary logistic regression models (multivariate) to determine significant predictors of testicular salvage in the overall cohort, as well as separately in the younger than eight and older than eight-year-old age groups. Variables at or approaching significance on univariate analysis (p<0.2) and clinically relevant variables were entered in the models. We included in the final models only variables that remained significant. All tests were performed using SPSS v 17.0 (Chicago, IL, USA) with statistical significance set a priori at α<0.05.

## RESULTS

We included 114 patients in the final analysis. Testicular salvage was possible in 63 patients (55.3%), and orchiectomy was performed in 51 patients. The mean age of the cohort was 11.3 ± 4.7 years. Thirty-five (31%) patients were transferred to the pediatric hospital after presenting in another ED, compared with 79 (69%) who presented primarily. In the entire cohort, median duration of symptoms prior to ED presentation was seven hours, and median time to operating room from first presentation was 3.33 hours. Testicular salvage rate was not different between patients who were transferred to our facility vs. those who presented primarily (60% vs. 53.2%, p=0.55). On univariate analysis, age, time to the operating room and duration of pain, was significantly different amongst patients who underwent orchiectomy vs. orchidopexy. Patient characteristics and results of the univariate analysis are listed in Table 1. Patients older than eight years of age were more likely to undergo orchidopexy than those younger than eight years (61.5% vs. 30.4%, p=0.01). Median duration of pain prior to presentation was higher in the orchiectomy vs. orchidopexy groups (42 vs. 4 hours, p<0.001). Patients who had symptoms for less than six hours were much more likely to undergo orchidopexy than those with symptoms for six hours or more (90% vs. 28.6%, p<0.001). Median time to the operating room from first presentation was higher in the orchiectomy group compared to the orchidopexy group (240 vs. 180 minutes, p=0.02). Ethnicity, insurance type, and laterality did not affect the testicular salvage rates.

Table 2 compares patients who were transferred to those who presented primarily. Transferred patients had longer median times to the operating room than primary patients (260 vs. 177 min, p<0.001). In regards to insurance status, transferred patients more likely to have Medicaid than those who presented primarily (40% vs. 19%, p=0.05); however, this was not statistically significant. Time of day, ethnicity, and age group did not affect transfer status.

On multivariate analysis of factors associated with testicular salvage, only duration of symptoms prior to presentation of less than six hours remained a significant predictor of testicular salvage (OR 22.5, p<0.001) (Table 3). Transfer status, age, time to operating room from first presentation, race, and insurance status were not significant predictors of testicular salvage. In a subgroup analysis of the patients ≥ eight years old, duration of symptoms was again the only predictor of testicular salvage (OR 22.5, p<0.001). In subgroup analysis of patients < eight years of age (n=23), no variables examined were predictive of testicular salvage.

## DISCUSSION

Our study showed that duration of symptoms prior to presentation was the most significant factor in testicular salvage overshadowing other factors such as age and transfer status. This was especially the case in older patients. Neither transfer status nor time to surgical exploration independently predicted testicular salvage. While transfer status did delay surgical exploration in our patient cohort, transferring a patient did not change overall outcome in our series. Nevertheless, delaying definitive surgical exploration may have resulted in orchiectomy in a small number of patients. In other words, transferring patients who already have relatively long duration of symptoms prior to presentation may result in orchiectomy. It is impossible to truly know whether transferred patients could have been salvaged had they not been transferred.

Children from surrounding areas are frequently transferred to our center, not only for treatment of urologic emergencies, but also for diagnostic tests. Lack of pediatric expertise at community treatment facilities, such as pediatric anesthesiology or pediatric radiology, may also necessitate transfer of pediatric patients. While such inter-hospital transfer is appropriate and essential in many cases, there is concern that it results in delay of care for time-dependent conditions such as testicular torsion.

Bayne et al.^1^ reported a series of 97 testicular torsion patients. Those investigators found that transfer delay in patients with potentially salvageable testes (i.e. those with symptoms less than 24 hours duration) puts those children at risk for orchiectomy. Patients who underwent orchiectomy also had longer pain duration and lived farther away from the hospital. Similar to our findings, the investigators reported that age was not an independent risk factor predicting orchiectomy, even though patients who received orchiectomy were younger than those who avoided orchiectomy.^1^

The clinical presentation of testicular torsion, especially in younger children may overlap other non-surgically managed processes, such as torsion of an appendix epididymis or appendix testis. While most adult men would be able to describe symptoms, such as sudden onset of pain, quality of pain, and duration of pain, pediatric patients are much less reliable historians. Because obtaining an accurate history is difficult, we have found that exploration for all acute scrotums may lead to many negative explorations. This has described by Lam et al.^5^ Therefore, most patients with the acute scrotum that present to our ED go immediately to ultrasound, a practice that has been endorsed by the American College of Radiology in their recommendations regarding imaging for acute onset scrotal pain.^6^

The current series failed to identify any predictors of testicular salvage in patients younger than eight years. Younger children may have difficulty communicating their symptoms to caregivers and may not have noticeable physical findings such as scrotal swelling or firmness until later in the course of their disease. Children who do not require as much assistance for bathing or using a toilet may not have close monitoring of their genitalia by their caregivers. Therefore, these young children may be the most vulnerable group, as they may not express their symptoms clearly. The duration of symptoms in this group may be even longer than what caregivers communicated in the patient’s history in the current series. In several studies, children with acute testicular torsion have symptoms other than scrotal pain and swelling, such as abdominal pain, nausea, and vomiting.^7^–^9^ It is crucial for providers to perform a focused genital examination for boys who present with abdominal pain, especially when patients may not be able to accurately describe their symptomatology.

Other researchers have shown that despite disease severity, children who are uninsured or have Medicaid insurance are more likely to undergo hospital transfer.^10^,^11^ We initially hypothesized that insurance status or socioeconomic factors may affect the rate of testicular salvage or transfer rates in our cohort. While insurance status did not impact the rate of testicular salvage, there was an alarming trend toward increasing transfer rates in patients with Medicaid compared to private insurance. While our study was not able to truly demonstrate statistical significance, this trend may very well be clinically relevant. Larger studies are needed to further characterize the effects of insurance on patient outcomes.

## LIMITATIONS

Our study has several important limitations, including the retrospective nature of the data. Chart reviews and historical accounts may have significant inaccuracies in regards to subjective information such as duration of symptoms. Charts and data from transferring institutions were not always available for review, making it difficult to know why patients were transferred. For the transferred patients, we could not determine if there was a local urologist available or if a local urologist had been contacted prior to initiating a transfer to our facility. We were also not consistently able to calculate distances from patients’ homes to the ED where they presented or if there was any correlation between home zip code and insurance type. Outcomes for those patients who were surgically treated at outside facilities instead of being transferred are also not known. We also may have had an inadequate sample size to detect significant differences resulting from insurance type. Specifically, the number of transferred patients was small compared to the number of patients who presented primarily, and the number of patients younger than eight years was small compared to patients older than eight years. Finally, we do not have long-term follow-up data for most of our patients, especially in terms of postoperative testicular viability in patients who underwent testicular salvage. Despite these important limitations, the current data reinforces conclusions in prior studies^1^,^4^,^14^ that delays in presentation and treatment are more likely to be associated with the need for orchiectomy.

## CONCLUSION

Time to presentation to the ED is the most important factor in predicting testicular salvage in patients with acute testicular torsion. Boys with acute scrotum should be referred quickly for emergency care to increase their chances of testicular salvage. Evaluation of abdominal pain should include a brief genital exam, especially in younger patients who may not be able to accurately describe their symptoms. Children and families should be educated on the seriousness of severe testicular pain and its consequences.

## Figures and Tables

**Table 1:** Patient characteristics, univariate analysis comparing testicular salvage.

	Orchiectomy n=51	Orchidopexy n=63	p-value
Median age (IQR), years	12 (4–14)	13 (12–14)	0.17
Age group			0.01
<8 years (n=23)	16 (69.6%)	7 (30.4%)	
≥8 years (n=91)	35 (38.5%)	56 (61.5%)	
Race			0.85
Hispanic (n=53)	24 (47.4%)	29 (52.6%)	
Other (n=57)	27 (45.3%)	30 (54.7%)	
Side			0.56
Left (n=69)	33 (47.8%)	36 (52.2%)	
Right (n=44)	18 (40.9%)	26 (59.1%)	
Insurance			0.09
Private (PPO/HMO) (n=48)	18 (37.5%)	30 (62.5%)	
Medi-cal HMO (n=37)	15 (40.5%)	22 (59.5%)	
Medicaid (n=29)	18 (62.1%)	11 (37.9%)	
Admission			0.55
Primary (n=79)	37 (46.8%)	42 (53.2%)	
Transfer (n=35)	14 (40%)	21 (60%)	
Median time to operating room (IQR), minutes	240 (163–320)	180 (140–240)	0.02
Median duration of pain prior to operating room (IQR), hours	42 (18–72)	4 (2–6)	<0.001
Duration of pain			<0.001
<6 hours (n=50)	5 (10%)	45 (90%)	
≥6 hours (n=63)	45 (71.4%)	18 (28.6%)	

*PPO,* preferred provider organization; *HMO*, health maintenance organization

**Table 2:** Comparison of primary and transferred patients.

	Primary	Transfer	p-value
Salvage			0.546
Orchiectomy (n=51)	37 (46.8%)	14 (40%)	
Orchidopexy (n=63)	42 (53.2%)	21 (60%)	
Age group			1.00
<8 (n=23)	16 (20.3%)	7 (20%)	
≥8 (n=92)	63 (79.7%)	28 (80%)	
Ethnicity			0.68
Hispanic (n=53)	36 (46.8%)	17 (51.5%)	
Other (n=59)	43 (41 (53.2%)	16 (48.5%)	
Time of day			0.42
Day (n=62)	45 (57%)	17 (48.6%)	
Night (n=52)	34 (43%)	18 (51.4%)	
Insurance			0.05
Private insurance (PPO/HMO) (n=48)	35 (44.3 %)	13 (37.1%)	
Medi-cal HMO (n=37)	29 (36.7%)	8 (22.9%)	
Medicaid (n=29)	15 (19%)	14 (40%)	
Median time to operating room (IQR), minutes	177 (140–229)	260 (220–360)	<0.001
Median duration of pain prior to operating room (IQR), hours	8.5 (3.8–48)	6 (2–24)	0.11

*PPO,* preferred provider organization; *HMO*, health maintenance organization

**Table 3:** Multivariate analysis predicting orchidopexy in the overall cohort and in patients ≥ 8 years old.

Duration of symptoms < 6 hours prior to ED presentation (≥6 = ref)	OR	95% CI		p-value
Overall cohort (n= 114)	22.5	7.69	65.83	<0.001
Age ≥ 8yr (n=91)	22.5	6.74	75.15	<0.001

*ED*, emergency department
